# The last journey, coming home before dying for migrant cancer patients: A case series

**DOI:** 10.1177/03008916251316175

**Published:** 2025-01-30

**Authors:** Luca Zambelli, Alberto Gigliotti, Clara Bianchessi, Simeone Liguori, Sergio Defendi

**Affiliations:** 1 Palliative Care, Pain Therapy, and Rehabilitation Unit, Fondazione IRCCS Istituto Nazionale dei Tumori, Milano, Italy; 2 Palliative Care Unit, ASST-Crema, Crema, Italy; 3 Medical Oncology Unit, ASST-Crema, Crema, Italy; 4 Palliative Care Unit, ASST Ospedale Papa Giovanni XXIII, Bergamo, Italy

**Keywords:** End-of-Life, migrant oncology patient, palliative and supportive care, patient repatriation, medical ethics in repatriation, cancer and migration

## Abstract

**Background:**

Global migration has led to an increasing number of migrant patients receiving cancer diagnoses in foreign countries. These individuals often experience worse outcomes due to advanced disease at diagnosis and limited access to specialized care. When palliative care becomes the primary option, many express a wish to return to their home country for End-of-Life care. However, no guidelines or care pathways currently address this sensitive issue.

**Case presentation:**

This case series describes three migrant patients who wished to return to their home countries for End-of-Life care. The first case highlights the critical role of early communication, administrative challenges, and disparities in palliative care availability. The second case demonstrates that even highly disabled patients can undertake a final journey if clinically stable, provided appropriate accommodations and support are in place. Both cases followed a similar framework: identifying a palliative care provider in the home country and having a Mini-Team member accompany the patient. In contrast, the third case underscores the difficulty of fulfilling this wish when clinical deterioration progresses rapidly, preventing repatriation

**Conclusion:**

Fulfilling the desire of migrant oncology patients to return to their home countries for End-of-Life care presents various challenges. These obstacles may arise from differences in national healthcare systems, administrative issues, and the patient’s clinical condition. It is crucial for the Mini-Team to identify this wish as early as possible to secure appropriate arrangements in the patient’s home country. Additionally, having a member of the Mini-Team accompany the patient during the journey can provide significant support.

## Introduction

In recent years, worldwide migration has continued to grow rapidly, with a growth rate of 62% since the start of the century, reaching 281 million in 2022. In this context, approximately 23 million valid residence permits were numbered in the European Union (EU). In the same year, Italy ranked as the fourth European country for the issuance of first residence permits to non-EU citizens, with a total of 337,788 new permits.^1^

This increase has led to a significant proportion of migrant patients receiving a new cancer diagnosis in foreign countries. Moreover, while improvements in cancer survival have been reported in high-income countries, such progress is still lacking in medium- and low-income countries.^2^ As a result, a growing number of patients with cancer diagnoses from medium- and low-income countries are migrating to high-income countries to access novel and/or expensive therapies.

Migrants and ethnic minorities have been identified as one of the most vulnerable groups in terms of health provision. When considering migrants with cancer, they are at a higher risk of being diagnosed with advanced disease and have less access to specialized medical care, resulting in worse outcomes.^3-4^ In addition, migrant patients report a lower compliance with oncological treatment compared to the rest of the population,^5^ likely due to low socio-economic conditions, language barriers and cultural differences.

Despite rising cancer survivorship, many patients still die from cancer, including migrants. Therefore, the need for palliative care among migrant cancer patients in foreign countries cannot be underestimated. Since palliative care is an approach that improves the quality of life of patients and their families,^6^ integrating of paradigms such as self-determination and patient empowerment within a multicultural care model is essential. When the focus shifts from oncological treatment to intervention aimed at improving the quality of life for patients with limited life expectancy, patients may express the desire to return to their home country for their End-of-Life (EoL) care.

Fulfilling this wish presents challenges for the palliative care and/or oncological teams. To date, no scientific society has addressed this issue in guidelines or Diagnostic and Therapeutic Care Pathways.

In this case series of three migrant cancer patients, we propose a framework to manage this challenging topic.

## Case description

Patient 1: A 45-year-old woman born in Somalia, who lived in Eritrea from childhood to 35-years old, was diagnosed with metastatic breast cancer while working in Italy. After multiple lines of oncological treatment, she was treated with capecitabine and vinorelbine until her performance status declined due to clinical progression, with worsened bone pain and the onset of hepatic failure, leading to daily nausea and confusion. The last reported blood exam showed increased total bilirubin (14.04 mg/dL), GGT (1895 UI/L), alkaline phosphatase (695 UI/L), and LDH (799 UI/L). As such, the patient was taken in charge by the local Home-Based Palliative Care Team. After 20 days of care, the patient’s symptoms were controlled, although her performance status steadily declined due to an increased fatigue, drowsiness and the development of a rapidly evolving peripheral edema. At this moment, she expressed the desire to return in Eritrea to die. While the entire Palliative Care Team was split on the possibility of her return home, given her poor performance status, her Mini-Team (i.e., the doctor and nurse responsible for her care) supported her desire.

Firstly, her Mini-Team helped her with the bank bureaucracy to secure the money to fly home. Then, they tried to find a hospital in Eritrea to take charge of the patient after her arrival, but it was not possible due to the absence of palliative care units in the country at the time.^7^ Therefore, a hotel room was booked in order to accommodate the patient for her EoL care. The next phase was to organize the flight and medical transport from her home to the airport.

Lastly, one member of the Mini-Team had to travel with her to manage the daily therapy and rescue medication during the flight. It is important to note that the patient was receiving through a daily elastomeric pump: morphine 100 mg, haloperidol 4 mg and metoclopramide 30 mg. The daily morphine use and its purpose had to be made explicit on a specific document. Still, an argument erupted at the border and the presence of the nurse from the Mini-Team was crucial to allow the drugs on the plane.

During the journey, the patient was mostly lethargic, anuric and the nurse from the Mini-Team administered a total of two rescue dose of parenteral morphine for uncontrolled pain.

The patient arrived in Eritrea, one week after she shared her wish, where she reunited with her father and her brothers. She died the following day.

Patient 2: A 35-year-old woman originally from Bolivia, was living in Italy as a housekeeper when she was diagnosed with primary central nervous system lymphoma. Despite surgery, radiotherapy, and multiple chemotherapy regimens, her condition worsened, leading to an ECOG PS of 4. She was subsequently admitted to the local hospice. Initially, she presented with minimal symptoms but began experiencing increasingly frequent episodes of agitation, characterized by screaming and crying. After one year of hospitalization, she remained completely bedridden and severely disabled. Her agitation had become refractory to neuroleptics and benzodiazepines, despite her otherwise stable clinical condition. Due to dysarthria, she struggled to communicate directly with her physicians, but she had previously expressed a strong wish to return to Bolivia to be with her son and parents. Given her stable clinical condition, her Mini-Team considered the possibility of repatriating her to Bolivia, where she had left behind her child and parents. As with the previous patient, the entire team was divided on this decision.

First, the Mini-Team contacted her parents to assess their ability to provide care. They also reached out to a hospital near her hometown to arrange for her continued care upon arrival. The Bolivian consulate was contacted to secure the necessary documentation, and the International Organization for Migration covered the flight costs for both the patient and a physician from her Mini-Team, who would administer rescue medication during the flight. Despite their request, the airline denied a first-class seat for the patient, which was sought due to her fragile condition.

During the journey, G (the patient) remained calm without agitation episodes.

Upon arrival in Bolivia, she was transferred to a local hospital as planned, where her agitation returned briefly. Five days later, she was discharged to her mother’s home under Home-Based Palliative Care. Remarkably, G did not experience further episodes of agitation after returning home. She passed away three years later.

Patient 3: A 63-year-old Romanian woman, who had been living in Italy for several years for work, was diagnosed with metastatic breast cancer. Throughout her illness, she frequently returned to Romania to visit her two sons and consistently expressed a strong desire to spend her final days in her home country.

As usual, she returned to her clinic in Italy as an outpatient to continue her chemotherapy regimen. During this visit, she reported new symptoms, including diplopia, difficulty walking, and impaired balance. Concerned about her worsening condition, her medical team ordered a CT-scan, which revealed the onset of leptomeningeal carcinomatosis. In response, her oncologists initiated a new chemotherapy regimen and planned to incorporate a palliative care team in her management. However, her condition deteriorated rapidly, leading to an emergency room admission. She was soon transferred to the Medical Oncology unit, where her Mini-Team, aware of her wish to return to Romania, immediately contacted her son. He arrived in Italy within a few days, hoping to bring his mother back to Romania.

Unfortunately, her condition rapidly worsened, making any attempt at transportation impossible. She passed away in the Italian hospital just a few days later, unable to fulfill her wish of dying in her home country.

## Discussion

To our knowledge, this is the first case series focusing on migrant oncology patients who wish to return to their home country for EoL care. All cases in this series followed a similar pattern. Once the Mini-Team identified the patient’s desire to return home, every effort was made to secure appropriate accommodation in their native country through a palliative care team or the patient’s family. This task presents significant challenges, as palliative care resources are not uniformly available worldwide,^8^ as demonstrated in Case 1.

A key step in these cases was the effort to assist the patient during the journey, given the potential need for medical interventions due to their severe clinical conditions. Interestingly, in all cases, the Mini-Team was more inclined to support the patient’s wish to return home, while other team members expressed uncertainty due to the patient’s clinical conditions. The coordinating physicians faced the complex task of balancing technical feasibility and medical needs with ethical considerations.

Case 1 underscores the importance of accurate medical information in predicting patient outcomes, which is crucial for determining the urgency of repatriation. Conversely, Case 3 illustrates how a patient’s condition can deteriorate rapidly and unexpectedly, potentially compromising the established plan and patient safety during transport.

Furthermore, Cases 1 and 2 revealed unique practical challenges, such as transporting an elastomeric pump with morphine or a bedridden patient in economy class. Although the patient from Case 2 did not experience symptoms during the flight, the possibility of developing refractory agitation was a significant concern.

A previous case series discussed the challenges of air medical repatriation for elderly patients with significant active illnesses.^9^ The authors emphasized the importance of a checklist approach before the journey to ensure that all relevant issues are addressed. This strategy aligns with our findings. Similar observations have been made in previous case reports.^10^ Given the absence of specific guidelines or diagnostic and therapeutic care pathways for such situations, developing a checklist could be valuable in managing these complex cases.

More high-quality studies are needed to better understand the needs of migrant patients at EoL and to explore potential solutions to this issue. It is especially important for palliative care societies to address this topic, standardize the management of such patients, and create a network to facilitate their final journey home.

## Conclusion

This case series highlights the complexities involved in fulfilling the wish of migrant oncology patients in returning to their home countries. The cases presented illustrate a potential approach to addressing the numerous medical, emotional, ethical, and administrative challenges that arise during this process. It is crucial for the Mini-Team to identify the patient’s desire to return home early on and assess the journey’s feasibility based on clinical conditions. If feasible, they should find a palliative care team in the patient’s home country. Otherwise, suitable accommodation that meets the patient’s medical needs must be arranged. Ideally, a Mini-Team member should accompany the patient during the journey to provide necessary medical assistance.
